# Understanding Defects in Amorphous Silicon with Million‐Atom Simulations and Machine Learning

**DOI:** 10.1002/anie.202403842

**Published:** 2024-04-18

**Authors:** Joe D. Morrow, Chinonso Ugwumadu, David A. Drabold, Stephen R. Elliott, Andrew L. Goodwin, Volker L. Deringer

**Affiliations:** ^1^ Inorganic Chemistry Laboratory Department of Chemistry University of Oxford Oxford OX1 3QR United Kingdom; ^2^ Department of Physics and Astronomy Nanoscale and Quantum Phenomena Institute (NQPI) Ohio University Athens Ohio 45701 United States; ^3^ Physical and Theoretical Chemistry Laboratory Department of Chemistry University of Oxford OX1 3QZ United Kingdom

**Keywords:** amorphous materials, computational chemistry, coordination defects, machine learning, solid-state structures

## Abstract

The structure of amorphous silicon (a‐Si) is widely thought of as a fourfold‐connected random network, and yet it is defective atoms, with fewer or more than four bonds, that make it particularly interesting. Despite many attempts to explain such “dangling‐bond” and “floating‐bond” defects, respectively, a unified understanding is still missing. Here, we use advanced computational chemistry methods to reveal the complex structural and energetic landscape of defects in a‐Si. We study an ultra‐large‐scale, quantum‐accurate structural model containing a million atoms, and thousands of individual defects, allowing reliable defect‐related statistics to be obtained. We combine structural descriptors and machine‐learned atomic energies to develop a classification of the different types of defects in a‐Si. The results suggest a revision of the established floating‐bond model by showing that fivefold‐bonded atoms in a‐Si exhibit a wide range of local environments–analogous to fivefold centers in coordination chemistry. Furthermore, it is shown that fivefold (but not threefold) coordination defects tend to cluster together. Our study provides new insights into one of the most widely studied amorphous solids, and has general implications for understanding defects in disordered materials beyond silicon alone.

## Graphical abstract

Machine‐learning methods and computational chemistry are combined to develop a comprehensive understanding of coordination defects in amorphous silicon. Fivefold‐connected atoms fall into three categories based on their chemical structure, and are found to cluster together, which can be explained based on energetic arguments.

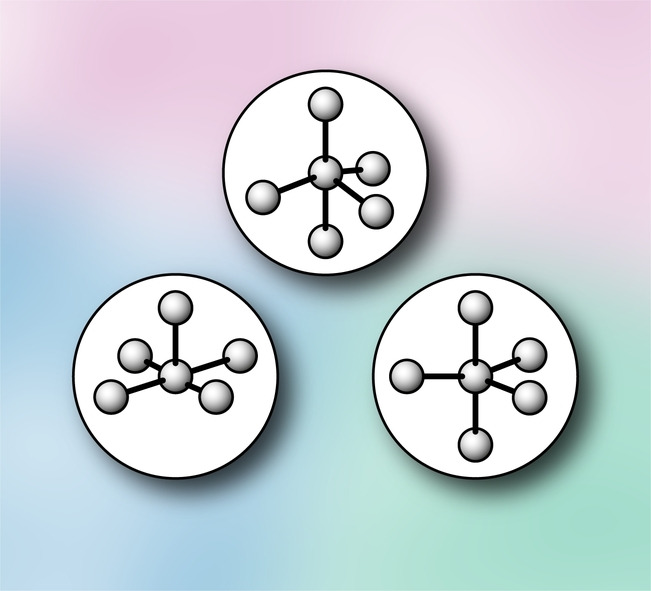

## Introduction

Amorphous silicon (a‐Si) is the textbook example of a disordered material, with a structure that overall resembles Zachariasen's concept of a continuous random network (CRN) of covalently bonded atoms.[^1^, ^2^, ^3^] Many interesting properties and phenomena relating to a‐Si have been discussed over the years: the hyperuniform nature of the disordered network,^[4]^ the transition between the high‐coordinated metallic liquid and the fourfold‐connected structure of a‐Si,^[5]^ its complex phase behavior under pressure,[^6^, ^7^, ^8^] and a tension–compression asymmetry in its mechanical properties.^[9]^ Many of those phenomena originate on the atomic scale, and fully understanding their origins in terms of chemical structure and bonding has long been a central research goal.

While the overall structure of a‐Si is based on fourfold‐connected atoms (*N*=4, where *N* denotes the number of bonded nearest neighbors), there is particular interest in those “defective” atoms that have fewer (*N*=3) or more (*N*=5) neighbors. In well‐relaxed structural models of a‐Si, *N* is easily determined: there is a clear minimum in the radial distribution function, separating the first (bonded) peak from the second (non‐bonded) one, at about 2.85 Å. Defining neighbors, and therefore coordination defects, is straightforward if the minimum in the distribution approaches zero; it becomes more ambiguous otherwise, which is most relevant to *N*=5 atoms. There is experimental evidence—for example, from spectroscopy—for the presence of point defects in a‐Si, emphasizing that its structure is more nuanced than a simplified all‐fourfold CRN description.[^10^, ^11^, ^12^, ^13^, ^14^, ^15^]

Computer simulations have long played a key role in studying amorphous networks, and a‐Si has been a prominent example.^[16]^ In the 1980s, models of a‐Si were created via melt‐quench molecular dynamics (MD) with the empirical Stillinger–Weber potential.^[17]^ The large concentration of coordination defects in the models obtained this way (≈20 %) permitted a discussion of the average structure of *N*=5 defects, as well as an analysis of the energetics predicted by the potential. Later studies, with increasingly fast computers, extended system sizes to hundreds of thousands of atoms with empirical potentials.[^18^, ^19^, ^20^] Equally, quantum‐mechanically based (density‐functional theory, DFT) MD studies on much smaller systems have provided important insights.[^22^, ^23^, ^24^] However, predictive DFT simulations of a‐Si beyond the few‐nm length scale remain out of reach, due to the long simulation times required and the cubic scaling of computational cost with system size. For example, a current “optimal” DFT‐MD‐based a‐Si structure contains 215 atoms,^[23]^ and the current limit for such types of MD simulations appears to be about 1,000 atoms.^[24]^ There is debate over the relative abundance of *N*=3 and *N*=5 defects, with spectroscopic evidence ambiguous[^25^, ^26^] and computational methodologies, such as ab initio MD^[21]^ and empirical potentials,^[27]^ tending to predict more 5‐fold defects.

Recent developments in machine‐learning (ML) based interatomic potentials have made it possible to prepare realistic, DFT‐accurate structural models of a‐Si of much larger size,[^8^, ^28^, ^29^, ^30^, ^31^] with a published million‐atom model reaching a cell length of about 27 nm.^[30]^ We have previously established that slow quenching from the simulated melt using ML potentials yields structural models whose characteristics agree well with existing experimental data.[^8^, ^28^, ^30^] There is increasing evidence that the local, per‐atom, energy predictions obtainable from atomistic ML (but not normally from DFT) are amenable to post hoc chemical interpretation: for example, we have shown that ML atomic energies in amorphous graphene can be used both to drive structural exploration and to analyze the resulting structures.^[32]^ Neural‐network models have been interrogated with regards to local energy contributions as well.[^33^, ^34^, ^35^] A recent study has shown that the density of ML atomic‐energy contributions can yield insights into complex solid‐state ion conductors.^[36]^

In a previous Communication in the present journal, we have shown that ML atomic energies can be used to discriminate three‐ and five‐coordinated atoms in a‐Si.^[29]^ We now build upon those pilot studies but expand on them vastly—for example, by analyzing a simulation cell containing a million atoms, of which several thousand are defective. We identify three structural prototypes for over‐coordinated atoms in a‐Si, allowing us to develop a “taxonomy” of defects in this canonical disordered material, and we explain the tendency for fivefold defects to aggregate via the strain that they induce on their atomic neighbors. Beyond silicon, our study paves the way for routine quantum‐accurate, million‐atom scale, ML‐driven simulations of rare events such as defect formation in functional materials.

## Results and Discussion

**Figure 1:**
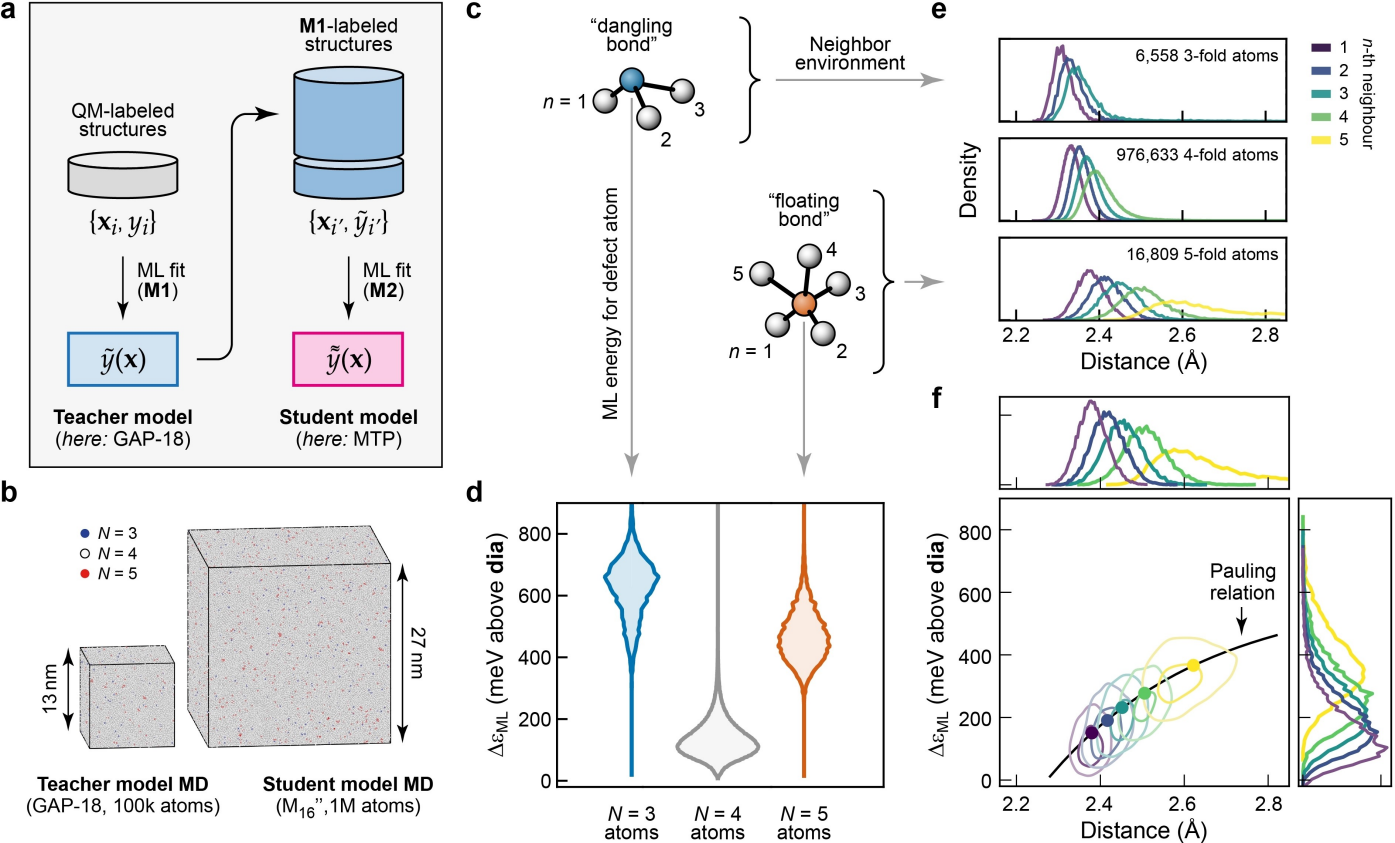
Defects in amorphous silicon from million‐atom simulations. (**a**) Schematic of the teacher–student approach to ML potential fitting described in Ref. [30]. A reliable, but relatively slow teacher model (**M1**) is used to generate a set of many more structures, to which the more specialized, but faster student model (**M2**) is then fitted. (**b**) Visualization of 100,000‐ (*left*) and million‐atom (*right*) structural models of a‐Si, drawn to scale for direct comparison. Panels (a) and (b) are adapted from Ref. [30], which was originally published under a CC BY licence (https://creativecommons.org/licenses/by/4.0/). (**c**) Schematic drawing of 3‐ and 5‐fold‐connected atoms, with neighboring atoms numbered in the order of their distance from the central atom. (**d**) Distributions of the ML‐predicted atomic energies, ϵ_ML_, of defects in the million‐atom model, shown separately for different nearest‐neighbor coordination numbers, *N*. All values are referenced to crystalline diamond‐type silicon, which is set as the energy zero. (**e**) Neighbor distributions, resolved according to 3‐, 4‐, and 5‐fold‐connected central atoms (separate axes) and their individual immediate neighbors, sorted by distance. (**f**) 2D correlation plot of the neighbor density for 5‐coordinated defects (as in panel e) versus energy (as in panel d), given separately for the immediate neighbors (*n*=1–5, purple to yellow). The black line describes a fit to a Pauling‐like relation,^[37]^ $\log\left(\;n\left(E\right)\;\right)=A\left(r_{c}-r\right)$ , where $n\left(E\right)\;$ is the bond strength defined in terms of neighbor local energies as ${(E}_{\max}-E)/{(E}_{\max}-E_{\min})$ , $r_{c}$ =2.38 Å is a characteristic radius (taken to be the mean minimum bond length in the structure), r is the bond length, and A is a fitting parameter with optimal value 1.17. Further details on the fitting function and choice of parameters are given in Figure S9.

Our simulations of a‐Si are based on a computational approach which we call “indirect learning” (Figure 1a),^[30]^ and which corresponds to teacher–student models for knowledge distillation that are more commonly used in ML research. The approach has been validated in our previous, more technical work,[^30^, ^38^] and we use it here to set the stage for an in‐depth analysis of the defects in a‐Si. Indirect learning involves using an accurate, trusted, but computationally slow ML potential (the teacher model) to train a second, much faster one (the student model). The latter enables simulations of accuracy and reliability on a par with that of the teacher (of the order of 10 meV per atom for amorphous Si vs. DFT), whilst requiring much less computational time, by a factor of about 1,000 in this case. A visual comparison between 100,000‐atom and 1 M‐atom models, drawn to scale in Figure 1b, illustrates the advantage of the approach. The structure factor and radial distribution functions are in very close agreement amongst experiment, teacher‐derived, and student‐derived structural models and the energetics are similar between student and teacher structural models for defects and 4‐fold atoms (Figure S1). Million‐atom simulation cells are necessary to systematically study defects that occur at the few‐percent level, and, crucially, to investigate “second‐order” processes, such as the interaction of defects with one another. Here, the simulation cell contains tens of thousands of examples of threefold‐ and fivefold connected defective atoms (Figure 1c). This simulation was affordable only because of the increased computational efficiency of the student model.

Perhaps the most serious assumption made with most current ML potentials is that the total energy of a system of atoms (a quantum‐mechanical observable) can be decomposed into a sum of local atomic energies (which are not observables).[^39^, ^40^] The major benefit of making this locality assumption is the ability to “machine‐learn” energetics independent of the system size, as well as the resulting linear scaling of computational cost with the number of simulated atoms. Although initially conceived for this purpose alone, there is increasing evidence that local energies have a physical relevance that can be useful for analysis beyond the mere construction of ML potentials,[^29^, ^36^, ^41^] and that their robustness can be quantified.^[39]^ We note that the idea of considering local contributions to the total energy in silicon has been pioneered based on empirical potentials,^[17]^ and our present ML‐based approach extends this type of thinking and places it on a more quantitative, DFT‐accurate basis.

Figure 1d shows the ML atomic energies in the 1 M‐atom model from Ref. [30], separated according to coordination numbers for the central atom. It confirms our earlier findings that *N*=3 atoms have distinctly higher energies on average than do *N*=5 ones.^[29]^ Note, however, that the distributions in Figure 1d result from a much larger dataset than in Ref. [29] and therefore do not include any broadening.

Figure 1e presents an analysis of the defects in a‐Si from an alternative, and somewhat orthogonal, purely structural perspective, by examining the distances to individual neighbors for each atom in the structure. As the coordination number of the central atom increases from 3 to 5, so too do the distances between bonded atoms, as is reasonable from a chemical perspective. The well‐defined 5‐th peak, and the even spacing between peaks for the 5‐fold distribution, indicate that a majority of such defects are best described as truly 5‐coordinated, rather than alternatively as [4+1] with a mostly non‐bonded 5‐th neighbor; the latter is what one would expect in tetrahedral amorphous carbon.^[46]^ The longer tail observed for the *n*=5 peak contains those more distant 5‐th neighbors that indeed are in this minor group of [4+1] environments. The typical bond lengths for *N*=3 and *N*=5 defects in our a‐Si model differ strongly from those for the non‐defective 4‐fold atoms in the rest of the structure, consistent with the observation that defects have significantly higher energies than do most bulk‐like atoms (Figure 1d).

In Figure 1f, we examine the correlation between those energetic and structural indicators, *viz*. the local energy of the neighbors of the 5‐fold atom on the *y*‐axis, and the distance of the respective neighbor from the central atom on the *x*‐axis. We find a striking, logarithmic relationship between these two quantities, which shows that the elevated local energy at 5‐fold defect centers is also delocalized onto the surrounding atoms with longer bonds to the defect. It is comforting that ML local energies, the physical meaning of which has been a source of debate, reproduce a well‐founded result from valence theory: the exponential dependence of bond strength on bond length. The equivalent correlation plots for *N*=3 and *N*=4 atoms (Figure S8) show a much tighter distribution of energies for the respective atomic neighbors.

The presence of some relatively short fifth‐neighbor contacts at distances below 2.6 Å (Figure 1e) invites the question whether there is a “typical” geometric structure of 5‐fold defects in a‐Si. In fact, this question has been studied for a long time. The term “floating bonds” has been used to describe defects consisting of silicon atoms with five bonded neighbors, initially introduced by Pantelides in 1986 as part of the first recognition of the importance of over‐coordinated atoms in a‐Si.^[25]^ The canonical structure of such a defect was described as a perfect tetrahedron with an extra fifth atom bonded directly opposite to one of the equivalent existing bonds. However, it was already noted that, in the amorphous phase, bond lengths and angles can vary considerably from such an idealized geometry.

**Figure 2:**
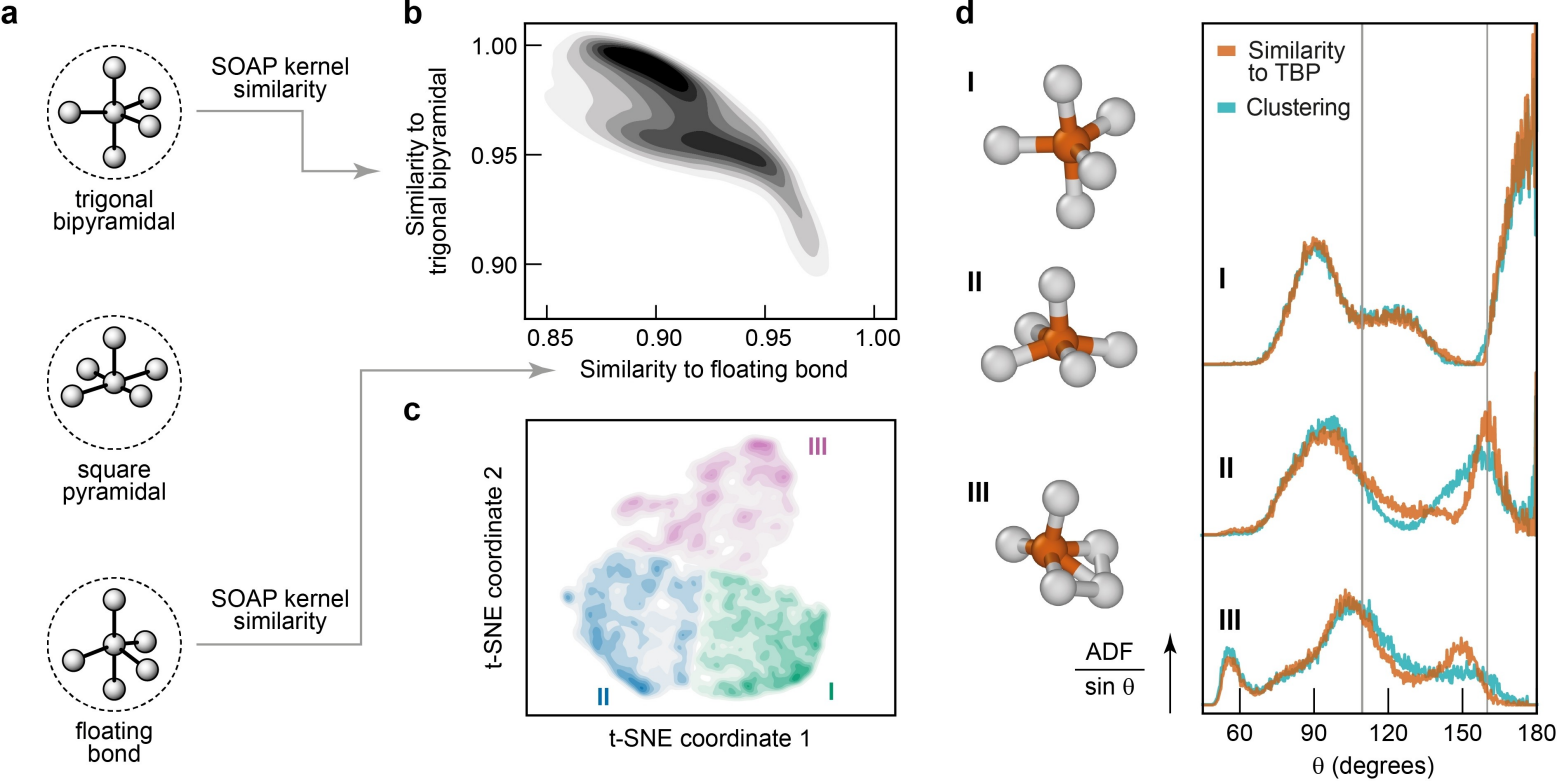
Categories of fivefold‐connected defects. (**a**) Schematic drawing of idealized trigonal bipyramidal (TBP), square pyramidal, and “floating‐bond” environments. The first two are shown in line with the established valence shell electron‐pair repulsion (VSEPR) model;[^42^, ^43^] the third is a tetrahedral environment with a fifth atom directly opposite a bond.^[25]^ (**b**) 2D plot of the SOAP kernel similarity of *N*=5 atoms in the a‐Si model to the idealized TBP and floating‐bond environments, respectively. The distribution of values for individual atoms is shown as a heat map. (**c**) Unsupervised classification of 5‐fold atoms. The full distance matrix, $D=\;\sqrt{2-2K}$ , is embedded in 2D with the dimensionality reduction algorithm t‐SNE,^[44]^ where K is the kernel matrix built from the similarity of each 5‐fold atom with every other 5‐fold atom. The bisecting *k*‐means algorithm is used to identify clusters **I–III**.^[45]^ See Figure S11 for more details. (**d**) Bond‐angle distribution function (BADF), scaled by sinθ , for atomic triples centered on all 5‐fold coordinated atoms. The BADFs are plotted separately for the three distinct categories of *N*=5 defects related to idealized structures respectively from top to bottom (as illustrated with selected examples of such configurations from the a‐Si model). The 5‐fold atoms are separated into these categories via two methods but with similar results: by comparison to the idealized structures of panels **a** and **b** via SOAP similarity (orange) and from unsupervised clustering (cyan). Vertical lines at the tetrahedral angle (109.5°) and at 160° are guides for the eye.

In Figure 2, we address this question with reference to the common prototypes for 5‐coordinated atoms that are known from structural inorganic chemistry. At the top of Figure 2a, we sketch the trigonal bipyramidal (TBP) and square pyramidal environments that one would expect from the valence shell electron‐pair repulsion (VSEPR) model[^42^, ^43^] that is frequently discussed in undergraduate chemistry textbooks, and also an idealized floating‐bond environment with a fifth bond directly opposite one of the bonds in a tetrahedral environment (bottom of Figure 2a).^[25]^

We took the 5‐fold defects in the 1 M‐atom structural model and evaluated their structural similarity to the three respective prototypes on a scale of 0 (dissimilar) to 1 (identical) via the Smooth Overlap of Atomic Positions (SOAP) kernel.^[47]^ In this analysis, we aimed to discriminate environments based on their bond angles and their respective similarity to the prototypical VSEPR configurations; we therefore re‐scaled all nearest‐neighbor distances to 2.5 Å, such that we examine only the angular distribution of atoms around each defect. A multimodal distribution of similarity values results, as displayed in Figure 2b. By dividing this distribution into three regions, we arrive at a classification of the 5‐fold defects in a‐Si as TBP‐like (**I**), square‐pyramidal‐like (**II**), or “floating bond”‐like (**III**).

A very similar classification is obtained using unsupervised ML[^48^, ^49^] in Figure 2c: dimensionality reduction followed by clustering. In this plot, the distance between points corresponds to their structural dissimilarity as measured by SOAP. The three‐lobed distribution, with higher densities of points at the outer edges, supports the identification of exactly three major classes of 5‐fold atoms.

We show bond‐angle distribution functions (BADFs) and accompanying representative example structures taken from the model in Figure 2d. We note that the BADFs in a‐Si have been closely linked to the Raman transverse‐optic peak width,^[50]^ to the exponential tails in optical‐absorption band edges, and to the through‐bond (topological) distance to over‐ and under‐coordinated atoms.^[51]^ Our million‐atom simulations allow us to derive finely resolved BADF plots,^[30]^ revealing immediately that the different categories have distinctly different shapes. The BADF for category **I** defects in Figure 2c directly reflects the angles in an idealized TBP environment: 180° between the axial atoms, 120° between equatorial atoms, and 90° between axial and equatorial atoms. The square‐pyramidal BADF (category **II**) has a fairly sharp distribution of angles at 160° and a broader peak at ≈100° angles. The designation of a “floating bond” (**III**), maximally dissimilar from the TBP (**I**), is less well‐defined structurally, but can be understood as originating from an ideal tetrahedron with an additional 5‐th atom approaching a face or edge, as originally suggested.^[25]^ In the BADF, this arrangement manifests in the following features: (i) the occurrence of ≈60° angles between the 5‐th atom and those neighbors that it approaches most closely; (ii) a corresponding closing of the ideal tetrahedral angle as these neighbors are displaced away from the 5‐th atom; and (iii) larger angles between the 5‐th atom and those on the opposite side of the tetrahedron. Fewer than half of all *N*=5 defects adopt a structure similar to the floating‐bond description.

The boundaries between the classes of 5‐fold defects, however, are somewhat fuzzy and this suggests an overlap of types of defect structures, rather than three entirely separate categories, as expected for such a strongly disordered amorphous structure. This observation is consistent with the low energy barriers between structural minima that are observed for 5‐fold complexes in molecular inorganic chemistry, which often exhibit fluxionality.[^52^, ^53^, ^54^] Further discussion of where we draw the boundaries between classes, along with partial pair correlation functions for defects, can be found in Figures S2 and S3.

**Figure 3:**
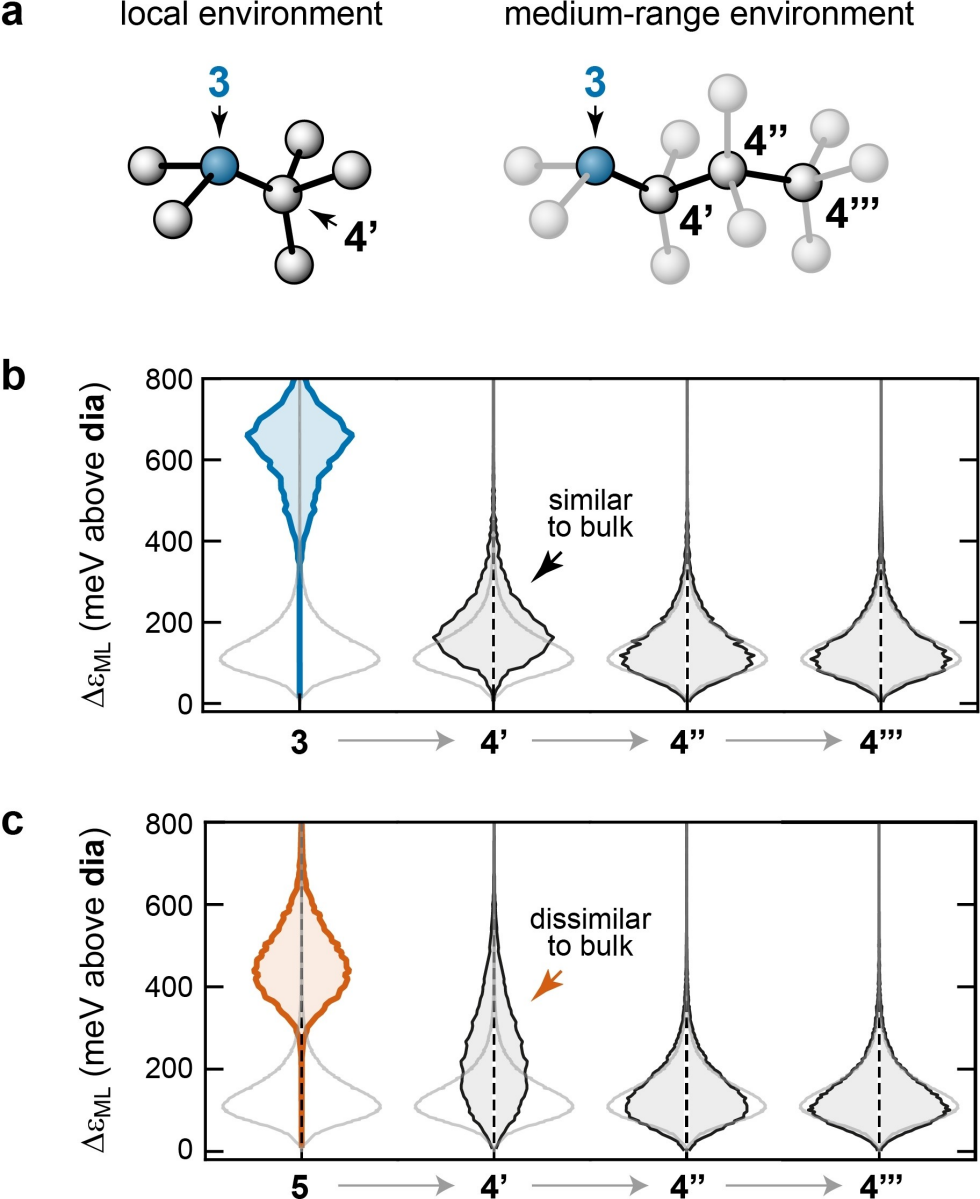
Locality of defect environments. (**a**) Schematic of the labeling system for local and medium‐range environments, based on the bond topology. A fourfold atom directly connected to a defect (here, to a 3‐coordinate atom) is labeled as 4′, a fourfold atom connected to that one is labeled as 4′′, and so on. (**b**) Distribution of ML local energies for *N*=3 atoms and their surroundings. The distribution for bulk a‐Si is shown in light gray. (**c**) Same but for *N*=5 atoms and their surroundings.

The correlation between bond length and local energy in Figure 1f had already suggested that the mechanical strain associated with 5‐fold defects extends over a longer range than the radius of the defect center itself. Figure 3 now provides a more detailed analysis of the atomic energies and their degree of “locality”–that is, their dependence on nearest‐, next‐nearest‐, and further neighbors. The locality of physical properties is of general interest for the development of atomistic ML models, because it determines directly the extent to which information can be captured by finite‐range models.^[55]^

We introduce some notation to describe the topological neighborhood of defective atoms, as sketched schematically in Figure 3a: 4‐fold coordinated atoms that are directly bonded to a defect center (i.e., to an under‐ or over‐coordinated atom) are referred to as 4′; 4‐coordinate atoms directly bonded to a 4′ atom, but not to a defect center, are referred to as 4′′; those bonded to a 4′′ atom, but not to any closer defect, are referred to as 4′′′. Atoms which are more topologically distant than the third neighbor shell, 4′′′, we merely call “bulk”.

Figure 3b indicates how, upon moving away from an *N*=3 defect atom, the atomic‐energy distributions quickly approach those of the bulk. The directly adjacent atoms, 4′, have a distribution that is similar to that of the bulk atoms (shown in light grey)—in other words, an *N*=3 atom does not seem to notably affect the atomic energies of its directly bonded neighbors. In contrast, Figure 3c shows that the 4′ atoms connected to an *N*=5 defect are much higher in energy on average. This suggests that the structural disturbance of defects in this case is less localized, and has a longer‐range effect. In both cases, the energy distributions for the 4′′ atoms and beyond are not notably affected by the presence of nearby defects.

The difference in the locality of the 5‐fold and 3‐fold defects has an interesting consequence: when the environment of each defect is considered, the locally averaged energy for 3‐fold and 5‐fold defects is 726 meV and 749 meV above the bulk, respectively, which indicates that 3‐fold defects are predicted to have a marginally *lower* formation energy, in contrast to what was found previously for the energies of the defect atoms themselves.^[29]^ Note that these sums take into account the clustering of 5‐fold defects described in Figure 5, which reduces the number of 4′ atoms per 5‐fold defect.

Why, then, are more 5‐fold defects observed in MD, despite their higher energy? This seemingly contradictory result can be rationalized as a consequence of either entropy or kinetics. The 5‐fold centers have considerably more flexibility than the relatively rigid pyramidal structure of 3‐fold defects, which implies 5‐fold defects may have higher entropy and hence a lower *free energy* of formation. 5‐fold defects are more similar to the structure of molten Si than are 3‐folds, so are likely to be more kinetically accessible during quenching. The difference in the local strain amongst 5‐folds and 3‐folds also leads us to advise care in interpreting a single local energy in the context of defects, as highlighted previously in the context of amorphous graphene.^[32]^ Local averages, which include physical strain induced by the defect and smooth out the assignment of local energies, appear to be more appropriate in drawing physical interpretations.

The structural and energetic landscapes of defects, even for seemingly simple crystalline materials, can be highly complex, and the exploration and understanding of those defects requires advanced computational techniques. For example, it was shown recently that relaxations of small‐scale defect models often determine incorrect (metastable) defect geometries, and that a more comprehensive exploration of the associated structural and energetic landscape is required even for seemingly simple inorganic crystals.[^56^, ^57^, ^58^] ML techniques have begun to be applied to defects in crystalline materials as well.[^59^, ^60^, ^61^] Here, we investigate the question whether there is a connection between defects in crystalline silicon, which are well‐studied, and the atomic environments of defects in the amorphous phase.

**Figure 4:**
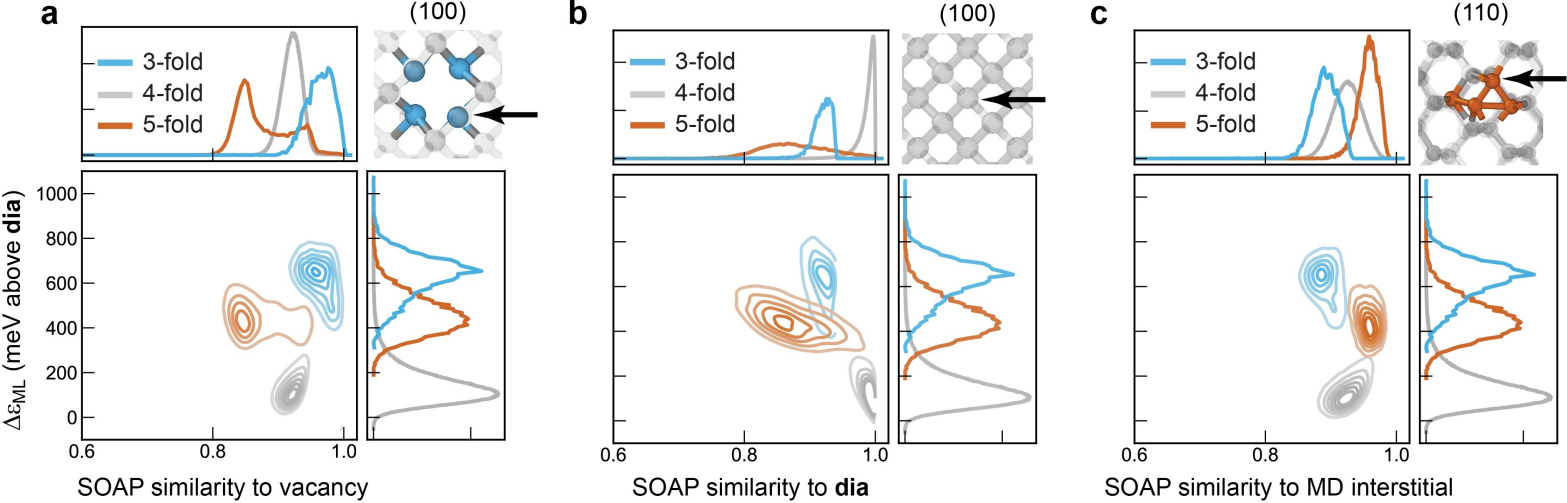
Connection with defects in crystals. (**a**) A 2D SOAP similarity–energy map for the structure obtained at a quench rate of 10^11^ K s^−1^, analyzing separately 3‐fold, 4‐fold, and 5‐fold coordinated atoms. The horizontal axis shows the structural similarity to the relaxed vacancy; the *N*=3 atoms (*blue*) are structurally the most similar to the vacancy; the *N*=5 atoms (*orange*) the least so. The vertical axis gives the ML atomic energy. The structure is visualized in the top right part, with the viewing direction given, and the arrow indicating the atom used for comparison. (**b**) Same for the perfect crystalline diamond structure, as in Ref. [29]. (**c**) Same for a snapshot from an MD simulation of an interstitial in the crystalline structure.

We have previously shown that 2D scatter plots of structural properties (specifically, the “diamond‐likeness”) and atomic energies are useful for understanding defects in a‐Si.^[29]^ We now extend this methodology by using it to compare defects in the amorphous form of silicon to different types of defects in the crystalline phase, as shown in Figure 4. The results are broadly in line with expectations: the 3‐fold defects are most similar to vacancies (blue in Figure 4a), 5‐fold defects are most similar to crystalline interstitials (orange in Figure 4c), and neither defect is very similar to the perfect crystal, as measured by the SOAP‐kernel similarity on the horizontal axis. The bimodal distribution in the similarity of 5‐fold defects to vacancies suggests that a minority of such defects, likely with two long bonds, could be considered as being related to vacancies. Note that the interstitial example was produced by equilibrating an idealized 10‐fold coordinated interstitial site in diamond‐type Si at 500 K. These simulations show that the structure of the crystalline interstitial, like the amorphous 5‐fold defect, is highly fluxional and is therefore difficult to understand via even several “typical” structures. Fluxionality of 5‐coordinate complexes is common in molecular systems because of the similar energies of different conformations, so it is perhaps unsurprising to observe a similar effect in an extended solid. In turn, this observation highlights the advantage of having many examples of individual defects in the million‐atom model available for comparison, as we have discussed in the context of Figure 3.

Our analysis in Figure 4 extends the long‐standing earlier assumption that amorphous materials contain structural building blocks of the corresponding crystalline phases.[^1^, ^62^] We therefore suggest that amorphous phases can be thought of as containing building blocks of crystalline phases *and of defects* in crystals.

**Figure 5:**
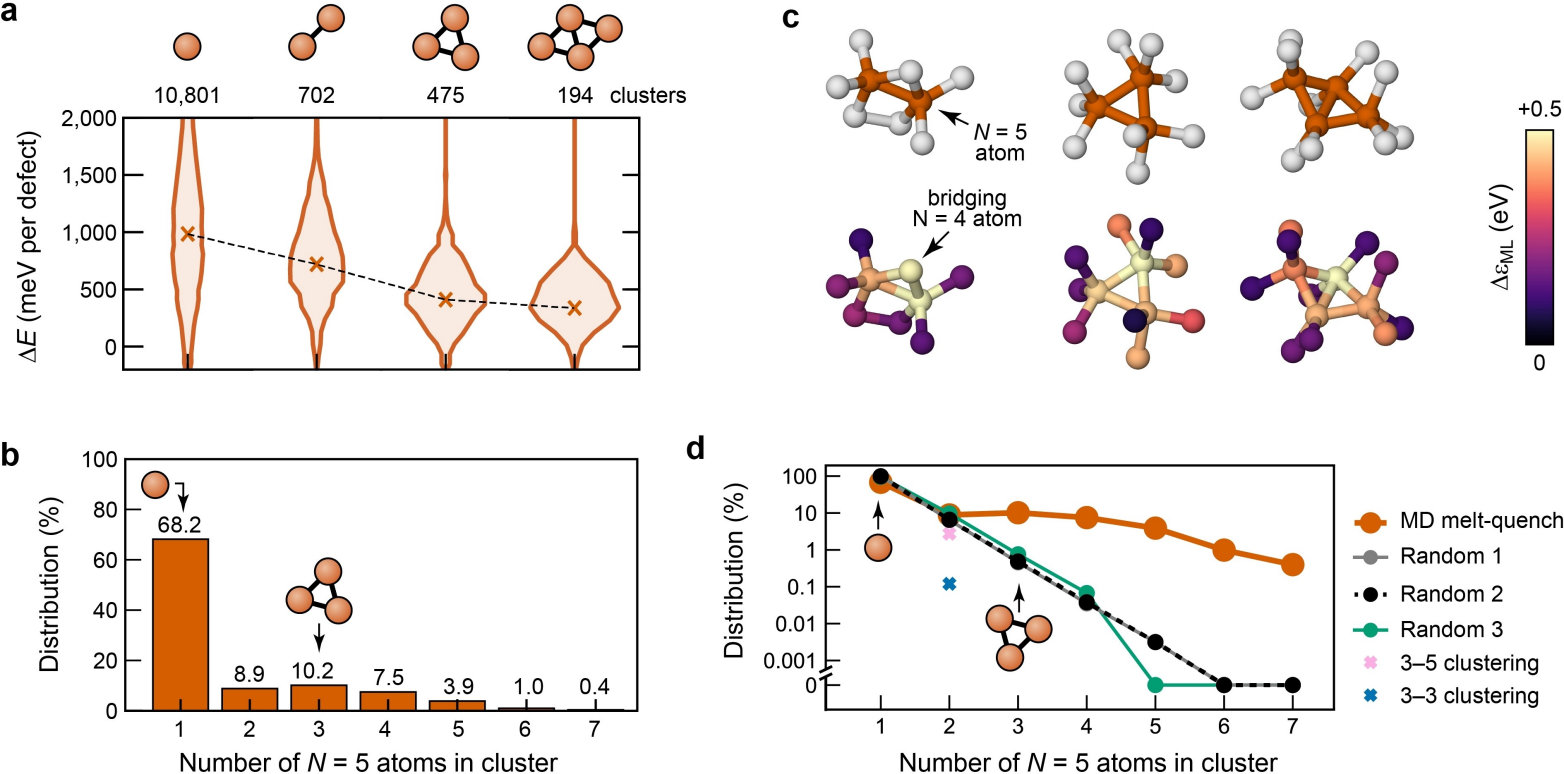
Clustering of defects in amorphous silicon. (**a**) ML‐predicted energy distributions for the most common 5‐fold defect clusters, with the number of occurrences of each structure directly above each histogram. Defect cluster energies are calculated by summing the individual atomic energies of defect cores and their immediate topological neighbors up to 3 bonds away relative to the mean a‐Si energy and are reported per coordination defect, as described in the main text. Crosses indicate the mean of each distribution; the dashed line is a guide for the eye. (**b**) Statistics for the number of occurrences of clustered 5‐coordination defects. (**c**) Examples of clustered *N*=5 defects of different sizes, color‐coded by coordination numbers of the atoms (*upper row*) and by their ML local energy above diamond‐type Si, ▵ϵ_ML_ (*lower row*). Note the high atomic energy of the bridging *N*=4 atom in the left part of the panel, indicated by an arrow. (**d**) Comparison of observed clustering of 5‐fold defects in the MD‐derived a‐Si model (orange, same data as in **b**) with three different idealizations of a random clustering distribution and the occurrence of 3‐fold‐5‐fold bonded pairs (pink cross) and 3‐fold‐3‐fold pairs (blue cross). Protocol 1 (gray) randomly labels 1.68 % of atoms (the 5‐fold defect concentration) in a perfect diamond structure as ‘defective’. Protocol 2 (black) randomly labels 1.68 % of atoms on the 4‐fold network of the a‐Si model as ‘defective’. Protocol 3 employs a bond‐switching algorithm to equilibrate the MD‐derived clustering distribution by moving 5‐fold centers randomly across the graph formed by atoms (nodes) and bonds (edges). This accounts for an increased likelihood of clustering purely due to the 5 bonds at 5‐fold‐coordinate atoms compared to the 4 bonds for most atoms. More details of the idealizations are given in the Supporting Information.

Although the majority of over‐coordinated defects in a‐Si exist as isolated *N*=5 atoms, we find a stronger tendency for those atoms to occur close together than would be expected for a random placement of defects in a CRN—i.e., the clustering that would be observed if 5‐fold defects were placed randomly without the influence of energetics. The energy distributions in Figure 5a, now evaluated for defects and their local environments up to 4′′′ sites, show that such clustering reduces the total average energy associated collectively with defect atoms and their immediate neighborhoods. This is largely a result of clustered defects having fewer nearest neighbors per 5‐fold defect. Neighbors of defects, on average, have an elevated energy compared to bulk a‐Si. To avoid the extensivity of the excess energy with surface area when referred to the ground‐state crystal energy, we reference the energies in Figure 5a to the average value for bulk a‐Si. In this way, the neighbors of a defect will only increase the energy of the defect cluster if they have an average energy above that of bulk a*‐*Si. Hence, we can converge the prediction of the cluster excess energy, so that it becomes independent of the number of neighbors considered, by including neighbors that are sufficiently distant from the defect center as to be typical of the bulk. In practice, this is achieved by including the 4′′′ atoms, *viz*. $\Delta E=\sum_{i}\left(E_{i}-\bar{E}\right)/n_{5}$ , where i indicates the topological classification of atom i and runs over 5, 4′, 4′′, and 4′′′; and $n_{5}$ is the number of 5‐fold atoms in the cluster. The widths of the distributions in Figure 5a get narrower with cluster size because larger clusters have a greater total number of 4′, 4′′ *etc*. neighbors (although fewer per 5‐fold atom). The relationship between probability and cluster size is approximately exponential (Figure 5b), with the exception of clusters of size 2, which are disfavored compared to 3‐membered clusters. 3‐fold defects are far less likely to cluster than 5‐fold defects: only 19 examples of 3–3 bonds were found (cf. Figure S12) and no clusters were found containing more than one 3–3 bond.

A possible qualitative explanation for this difference is provided by the examples in Figure 5c: in the first case, a pair of *N*=5 atoms is connected via a bridging *N*=4 atom which in itself has a rather high atomic energy (highlighted by an arrow), consistent with the strain formed in the associated three‐membered ring. In the larger fragments shown in Figure 4c, there are still three‐membered rings, but they are now formed by three and four *N*=5 atoms clustering together, respectively. In these cases, the directly‐connected *N*=4 atoms have lower energies. The clustering of 5‐coordinate defects can be interpreted as causing a reduction in the defective “surface area”.

## Conclusions

Our analyses support a comprehensive picture of defects in amorphous silicon. On the one hand, 3‐fold connected “dangling‐bond” defects are high in energy and do not strongly affect their surroundings. On the other hand, 5‐fold connected defects are associated with a broad range of possible structures^[29]^ which we can understand, at one extreme, as being similar to a trigonal bipyramid with an even distribution of bond lengths, and at the other extreme, as being similar to the floating‐bond description applied previously. 5‐fold defects also have an extended influence on their surroundings, reaching beyond their immediate atomic neighbor environment, which explains their observed tendency to cluster together.

Beyond silicon, our study has more general implications for materials modeling. Defects and impurities in solids occur at a concentration of a couple of percent at most, yet they are highly consequential for the performance of functional materials. The structure and chemical bonding of these defects likely differ markedly from the bulk, requiring a quantum‐mechanically accurate description of the potential‐energy surface associated with defect formation. In this work, we showcased how ML interatomic potentials can be used to study rare events in a systematic way, and how ML‐predicted atomic energies can explain the stability of defect environments in one of the canonical amorphous materials, *viz*. a‐Si. Qualitatively, this means a step away from idealized CRN models or small‐scale simulation systems which (necessarily) contain only a handful of defects, moving towards a fully realistic description of the amorphous state.^[63]^ In the future, we envisage similar ML‐driven studies of defects and defect complexes in a wide range of functional materials.

## Conflict of interests

The authors declare no conflict of interest.

1

## Supporting information

As a service to our authors and readers, this journal provides supporting information supplied by the authors. Such materials are peer reviewed and may be re‐organized for online delivery, but are not copy‐edited or typeset. Technical support issues arising from supporting information (other than missing files) should be addressed to the authors.

Supporting Information

## Data Availability

Data supporting the present study are publicly available at https://doi.org/10.5281/zenodo.10794053.
