# Supplementary material for: Understanding Defects in Amorphous Silicon with Million‐Atom Simulations and Machine Learning
Source: Angew Chem Int Ed Engl. 2024 Apr 18;63(22):e202403842. doi: 10.1002/anie.202403842 (PMC11497335; doi:10.1002/anie.202403842)
Supplement: Supplementary file 1 — Supporting Information [file ANIE-63-e202403842-s001.pdf]

Angewandte Chemie
Eine Zeitschrift der Gesellschaft Deutscher Chemiker

Supporting Information

Understanding Defects in Amorphous Silicon with Million-Atom Simulations and Machine Learning

J. D. Morrow, C. Ugwumadu, D. A. Drabold, S. R. Elliott, A. L. Goodwin, V. L. Deringer**

Supporting Information for

Understanding Defects in Amorphous Silicon with Million-Atom Simulations and Machine Learning

Joe D. Morrow,[a] Chinonso Ugwumadu,[b] David A. Drabold,[b] Stephen R. Elliott,[c] Andrew L. Goodwin[a,]* & Volker L. Deringer[a,]*

[a] *Inorganic Chemistry Laboratory, Department of Chemistry, University of Oxford, Oxford OX1 3QR, UK*

[b] *Department of Physics and Astronomy, Nanoscale and Quantum Phenomena Institute (NQPI), Ohio University, Athens, Ohio 45701, United States*

[c] *Physical and Theoretical Chemistry Laboratory, Department of Chemistry, University of Oxford, Oxford OX1 3QZ, UK*

* andrew.goodwin@chem.ox.ac.uk; volker.deringer@chem.ox.ac.uk

Computational Methods

Teacher–student ML potentials

We generate fast and robust interatomic potential models by using one ML model to "teach" another. This idea is related to knowledge distillation in neural networks, albeit defined more generally. In Ref. [S1], we demonstrated it for a kernel-based method, *viz.* the Gaussian approximation potential (GAP) framework,[S2] as the teacher model, and a linear-fitting technique, viz. the moment tensor potential (MTP) approach,[S3] as the student model. We used the indirectly-learned M_{16}'' model[S1] to generate structures via MD, and we used local energies predicted by the teacher GAP model[S4] for analysis. Using the M_{16}'' local energies gave qualitatively similar results. The difference in total energy between teacher and student methods is 3 meV per atom, with a root-mean-square difference of 48 meV for local energies. This difference in local energies is of a similar size to that achieved by training directly on local energies derived from GAP models for carbon and silicon.[S5,S6]

Molecular dynamics

MD simulations and local-energy evaluations were carried out using the LAMMPS software,[S7] interfaced to the MTP[S3] and GAP[S2] codes, respectively. The timestep was 1 fs. In Ref. [S1], melt–quench simulations were conducted using the same variable-rate protocol as described in Ref. [S8], that is:

1. Melting a random initial structure at 2500 K for 20 ps
2. Equilibrating the resulting liquid at 1500 K for 100 ps
3. Quenching at a variable rate: 10^{13} K s^{-1} between 1500–1250 K, 10^{11} K s^{-1} between 1250–1050 K over which vitrification takes place, back to 10^{13} K s^{-1} between 1050–500 K for a total cooling time of 2.08 ns, before a relaxation with conjugate-gradient (CG) descent.

The same type of analysis of defects performed herein on the structure cooled at 10^{11} K s^{-1} (taken from Ref. [S1]) was performed on a separate 1M-atom a-Si structure produced by annealing a rapidly quenched structure (10^{13} K s^{-1} throughout the temperature range 1500–500 K) at 850 K for 3 ns before cooling back close to 0 K at 10^{13} K s^{-1} and relaxing by CG descent. The results for the annealed structure, which are equivalent to those in the main article, may be found in Figures S6–S7 and show that our conclusions are robust with respect to the precise number of defects and the path to reaching them.

Electronic-structure analysis for validation

We analyzed with DFT the electronic structure of a small-scale (512 atom) structural model made in the same way as the million-atom model to further validate structures produced with the ML potential. A clear energy gap between valence and conduction bands and exponentially shaped Urbach tails are indicators of high-quality structural models of a-Si.[S9] In our model, Urbach tails are evident and dangling bonds produce electronic states near the middle of the gap (Figure S4). Floating bonds in these models, which are thought to play a role in charge-carrier transport,[S10] do not produce localized states in the gap, and as such would not be observed in an electron spin resonance experiment, similar to observations in Ref. [S11]. However, we observe that tail states are distributed among 4′ atoms in the vicinity of the floating bonds (Figure S5). This analysis suggests that only the dangling-bond sites would yield an electronic signature in the gap,[S12] for which the concentration is low compared to other MD-derived structures at 0.7%.[S13] Hence, the electronic properties of the million-atom model are consistent with other high-quality structural models. Further details of the electronic-structure calculations, including analysis of the ultra-large structure with a tight-binding Hamiltonian, are provided in Figures S4–S5 and their captions below. As an aside, we note that regions of strain defined by a 5-fold-coordinated atom and its immediate 4′ neighborhood are rather more mobile than the atoms themselves, which allows the strained regions to diffuse through the

structure at an appreciable rate even after vitrification has slowed atomic diffusion. The time-average of this motion could further contribute to the exponential Urbach tails.

Calculation of local averages for defect formation energy

A rough estimate of the energy of a 5-fold defect, including its environment, can be made using the mean values of distributions in Figure 3, after subtracting the bulk mean energy, and counting the approximate number of neighbors (neglecting the possibility of shared neighbors):

331 meV (5-fold) + 5 × 104 meV (4′) + 15 × 7 meV (4″) + 45 × 0.07 meV (4‴) = 964 meV

A similar calculation for 3-fold defects gives 772 meV.

Summations of the local-energy data using the following pair of methods are consistent with this estimate. Figure 5a of the main text includes 5-fold defects among the 10,801 'isolated' cases provided there are no direct bonds between two 5-folds. An isolated 5-fold's environment is then constructed by searching for all surrounding 4′ atoms whilst ignoring any other defects, then 4″s, and finally 4‴s. This encompasses cases such as a bonded 5–4′–5 triple, where the second 5-fold would be ignored. The average energy of 'isolated' 5-fold atoms under this definition is 984 meV.

An alternative definition of 'isolated' considers only 5-fold atoms for which no other defects occur up to the surrounding 4‴ shell so no other 5-folds are found within 3 bonds. There are fewer (3,953) of such 5-fold defects in the 1M-atom structure, with an average energy of 950 meV.

Across almost all defects (> 97%), using the second definition, the average energy of 5-folds and their environment, including those that cluster, is 749 meV. This value compares with average energies for 3-fold atoms of 726 meV. The remaining 3% were excluded because these rare cases involve, for example, 2-fold and 6-fold connected atoms, or more complicated clusters involving 3- and 5-fold coordination which are complex to handle systematically.

Idealizations of random clustering distributions

The clustering distributions of the following models are included in Figure 5d of the main text to illustrate the increased probability of 5-folds clustering.

Random protocol 1: 16,809 atoms in a 1M-atom perfect diamond structure (the same defect concentration as in the MD structure) were labeled as 'defects', without disturbing their positions or bonding topology.

Random protocol 2: 16,809 random atoms in the amorphous 1M-atom structure were relabeled as 'defects' using the existing bonding topology defined by a 2.85 Å cutoff. The majority of these are, statistically, 4-fold connected atoms.

Random protocol 3: a bond creation/deletion procedure takes the original clustering distribution of the MD-derived model and randomizes it by walking 5-fold defects across the graph initialized by the bonds in the structure (2.85 Å cutoff). The atomic positions are ignored, with only the bonding topology modified at each step as follows:

1. A 5-fold connected atom is selected at random for movement (atom $\boldsymbol{i}$)
2. A 4-fold connected target is selected at random to become the new 5-fold one under the constraint that it must be exactly 3 bonds away from atom $\boldsymbol{i}$ (atom $\boldsymbol{l}$)
3. Atoms $\boldsymbol{j}$ and $\boldsymbol{k}$ are selected randomly to form a path from atom $\boldsymbol{i}$ to atom $\boldsymbol{l}$ under the constraint that both must not be neighbors of either $\boldsymbol{i}$ or $\boldsymbol{l}$

The bond $\boldsymbol{i}$–$\boldsymbol{j}$ is deleted and bond $\boldsymbol{j}$–$\boldsymbol{l}$ is formed, which moves the 5-fold across the graph by 3 bonds. This procedure can be repeated until the cluster distribution is equilibrated, giving a random distribution in green in Figure 5 after 1.65 million moves (approx. 100 moves per 5-fold center).

Supplementary Figures

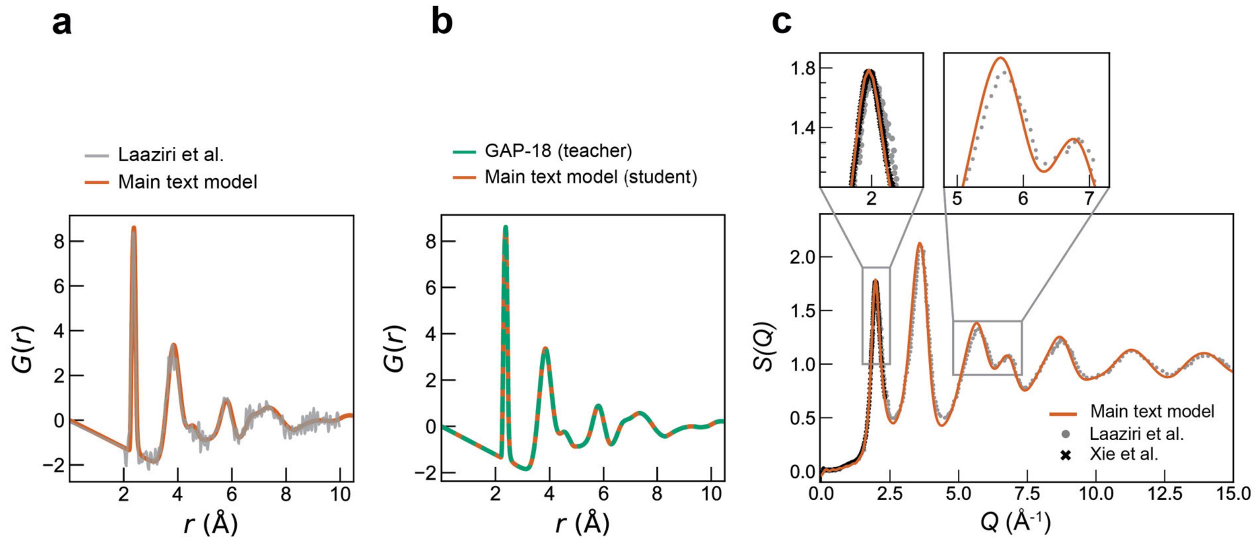

Figure S1. Comparison of structural indicators for 1M-atom model with experiment. (**a**) Total radial distribution function, *G*(r), for the 1M-atom model of the main text compared to the experiment from Ref. [S14]. (**b**) *G*(r) for the same structural model compared to the 100,000-atom model produced by GAP-18,[S4] which is the "teacher" potential as described in Ref. [S1]. (**c**) Structure factor, *S*(Q), for the 1M-atom model (orange) compared with experiments from Refs. [S14] (gray) and [S15] (black). The difference in total energy between teacher and student methods is 3 meV per atom, with a root-mean-square difference of 48 meV for local energies.

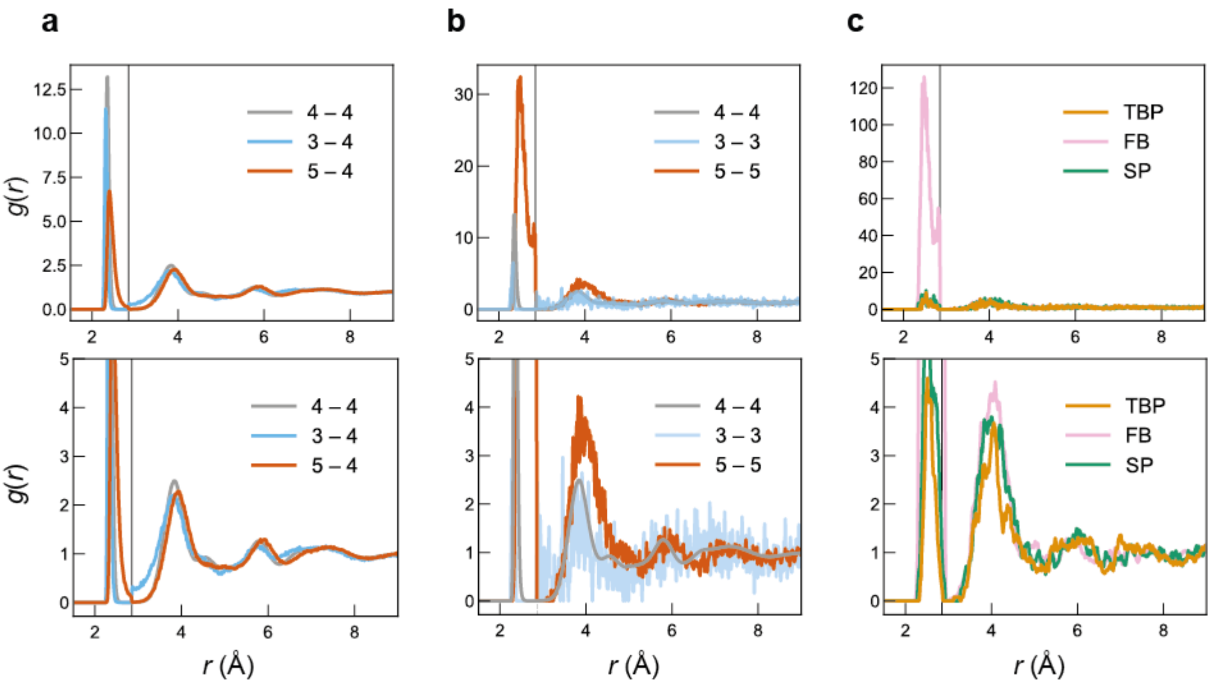

Figure S2. Pair correlations for defect atoms. In all columns, we show radial distribution functions, $g(r)$, in full (upper panels) and magnified (lower panels). (**a**) Correlations between each of 3-, 4-, and 5-fold connected atoms and 4-fold connected atoms. (**b**) Correlations between atoms of the same connectivity. (**c**) Correlations between 5-fold connected defects, separated by classification into trigonal-bipyramid-like (TBP), "floating bond"-like (FB), and square-pyramidal-like (SP) as defined in Figure 2 of the main text. Vertical lines indicate the radial cut-off used to define coordination numbers.

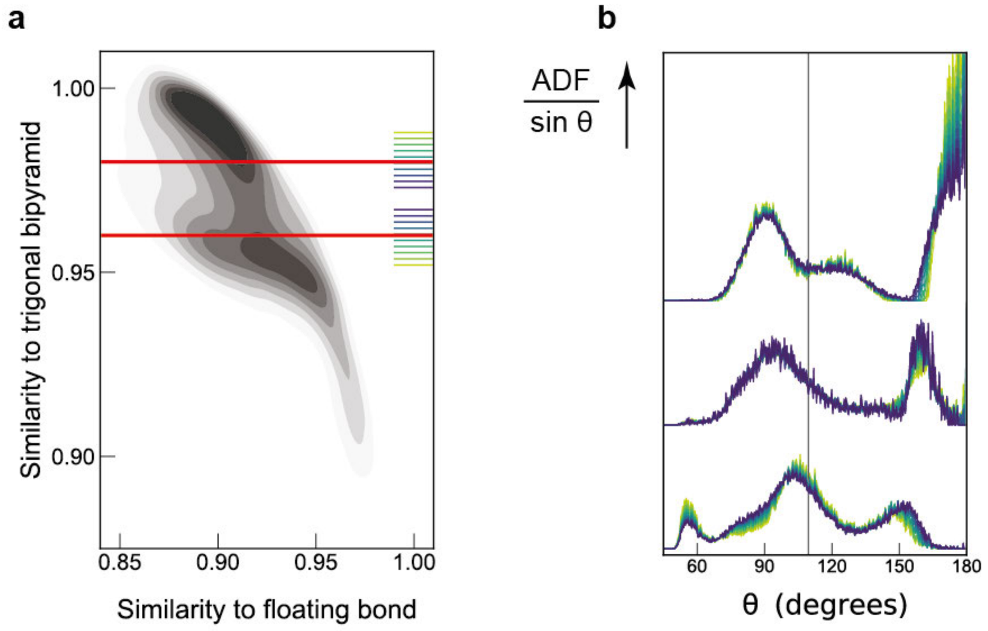

Figure S3. Classification methods for defects. (**a**) SOAP similarity values used for defining the three classes of fivefold defect (red lines) in Figure 2 of the main text. Structures with a similarity greater than the upper red line are categorized as trigonal-bipyramid-like, those between the lines as square-pyramidal-like, and those below as "floating bond"-like. (**b**) Sensitivity of ADFs to similarity cut-off values. ADFs are shown in colors purple to yellow corresponding to a range of similarity values in panel a of the same color. The form of the ADF is quite insensitive over a wide range of similarity values; therefore the results in Figure 2 are not strongly affected by the exact cut-off value for the similarity to distinguish classes.

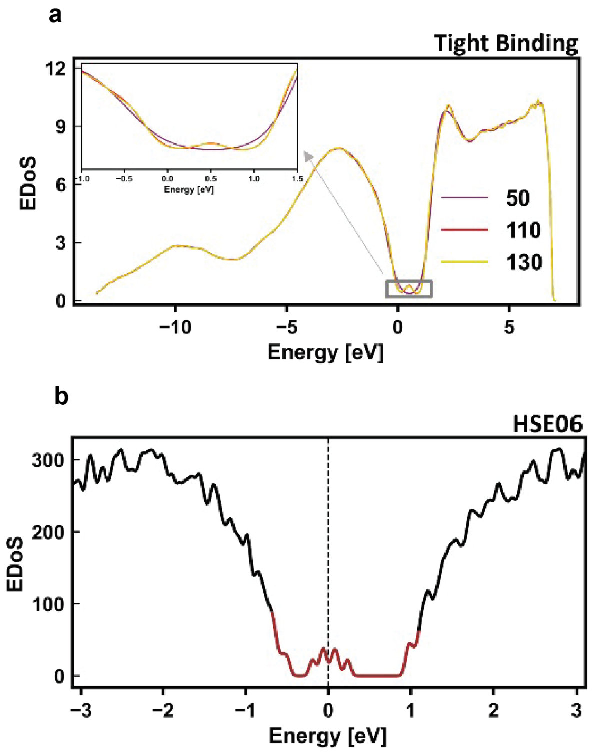

Figure S4. Electronic-structure analysis for validation of a-Si models. (**a**) Electronic density of states (EDoS) for the 1-million atom a-Si model discussed in the main text, computed using the tight-binding methodology of Ref. [S16]. The coloring of the lines refers to the number of moments used in the maximum-entropy reconstruction. See Ref. [S17] for a summary of the methodology and an example of a DOS computation on a similarly large (albeit crystalline) Si structure. (**b**) EDoS for a 512-atom a-Si model computed with the HSE06 hybrid functional.[S18,S19] The 512-atom model is constructed using the same protocol as for the 1-million atom model, and the two models exhibit gaps of similar width. The Fermi level is shifted to E = 0 eV. The mid-gap region is highlighted in brown and discussed further in Figure S5.

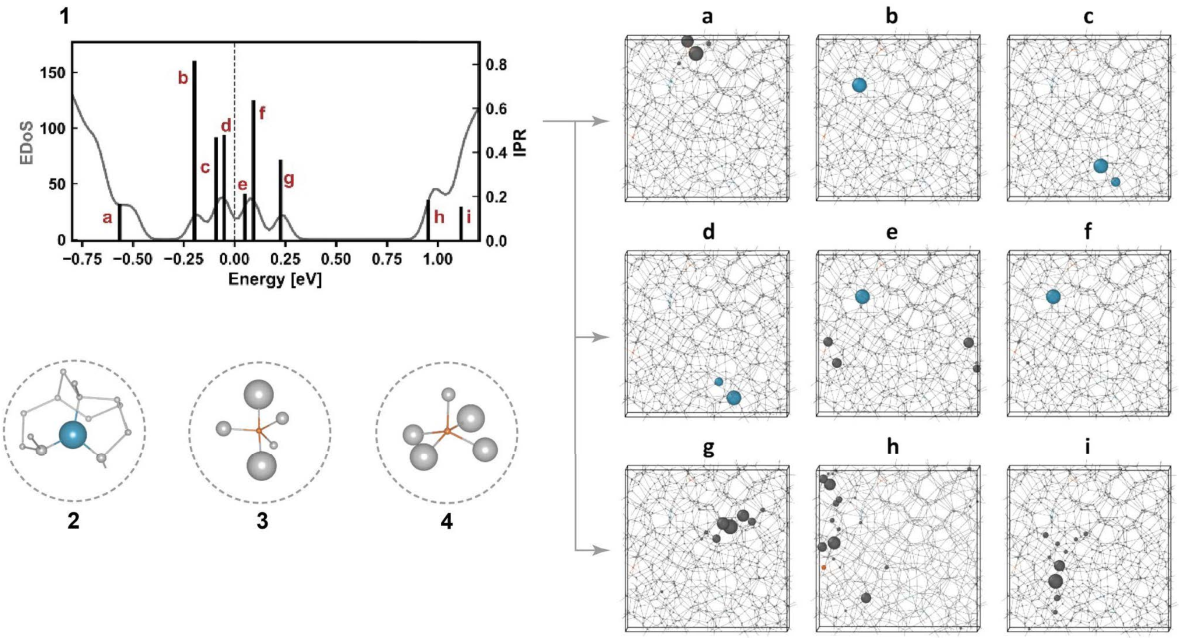

Figure S5. (**1**) Localized states within the energy-gap region (brown region in Figure S4b) obtained for the 512-atom structural model. (**a–i**) Projection of localized states onto the 512-atom model, labeled **a–i** in panel **1**. (**2–4**) Localization environment for mid-gap and tail states of defects and their neighbors. The sizes of the spheres centered on atoms indicate the degree of localization, with 3-, 4-, and 5- fold atoms shown in gray, blue, and orange colors respectively, for both **2–4** and **a–i**. States near mid-gap are localized exclusively on dangling bonds, consistent with earlier electronic-structure computations in Ref. [S12]. The environment of the localized 3-fold atom in **b**, **e**, and **f** is shown up close in **2**. The valence tail state **a** is distributed among 4-fold atoms that are directly connected to floating bonds in a tetrahedral environment with a fifth atom directly opposite a bond (as described in the main text). The environment of **a** is illustrated in more detail in **3**. The electronic state immediately above the Fermi level, denoted as **e** in panel **1**, consists of a linear combination involving a 3-fold atom and 4-fold atoms directly connected to a 5-fold atom in a square pyramidal environment—also discussed elsewhere.[S20] The localization environment of **e** is essentially a mixture of environments **2** and **4**. The conduction tail state **h** is primarily localized on 4-fold coordinated atoms near the same 5-fold atom in the square pyramidal environment of **4**. Our 512-atom cell may be expected to produce representative defects but is not necessarily exhaustive due to its modest size.

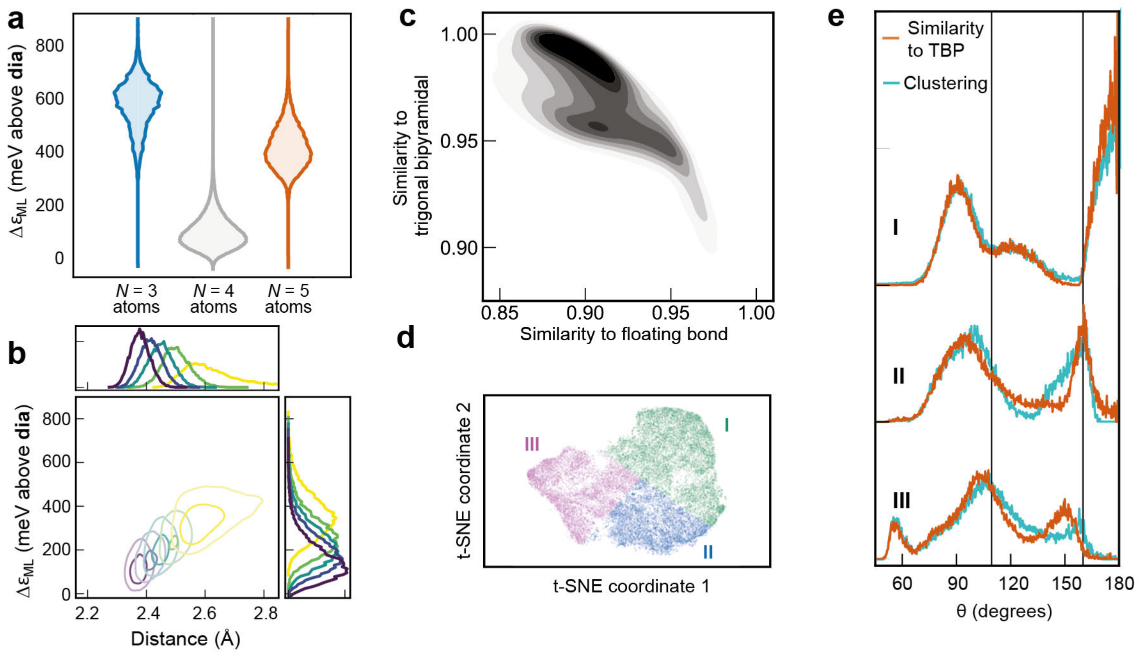

Figure S6. An alternative 1-million atom structure from rapid quenching and annealing. We repeat analyses as in the main text for a separate, entirely uncorrelated 1M atom structure, which was produced with a slightly different protocol: rapid cooling at 10^{13} Ks^{-1} followed by a long annealing period of 2 ns at 840 K. This structure has a similar number of 5-fold atoms as the structure discussed in the main text, which was derived from slower cooling at 10^{11} K s^{-1}. (**a**) Distributions of the ML atomic energies, ε_{ML}, of defects in the model, shown separately for different nearest-neighbor coordination numbers, *N*. (**b**) Two-dimensional correlation plot of the neighbor density for 5-coordinated defects (as in panel **e**). (**c**) 2D plot of SOAP kernel similarity of $N = 5$ atoms in the a-Si model to the idealized TBP and floating-bond environments, respectively. The distribution of values for individual atoms is shown as a heat map. (**d**) Unsupervised classification of 5-fold atoms. The full distance matrix, $\mathbf{D} = \sqrt{2 - 2\mathbf{K}}$, is embedded in 2D with the dimensionality-reduction algorithm t-SNE,[S21] where **K** is the kernel matrix built from the similarity of each 5-fold atom with every other 5-fold atom. Bisecting *k*-means is used to identify clusters **I**–**III**. (**e**) Bond-angle distribution function (BADF) plots, scaled by $\sin\theta$, for atomic triples centered on all 5-fold coordinated atoms. The BADFs are plotted separately for the three distinct categories of 5-fold defects related to idealized structures respectively from top to bottom (as illustrated with selected examples of such configurations from the a-Si model). The 5-fold atoms are separated into these categories via two methods but with similar results: by comparison to the idealized structures of panels **a** and **b** via SOAP similarity (orange) and from unsupervised clustering (cyan). Vertical black lines at the ideal tetrahedral angle (109.5°) and at 160° are guides for the eye.

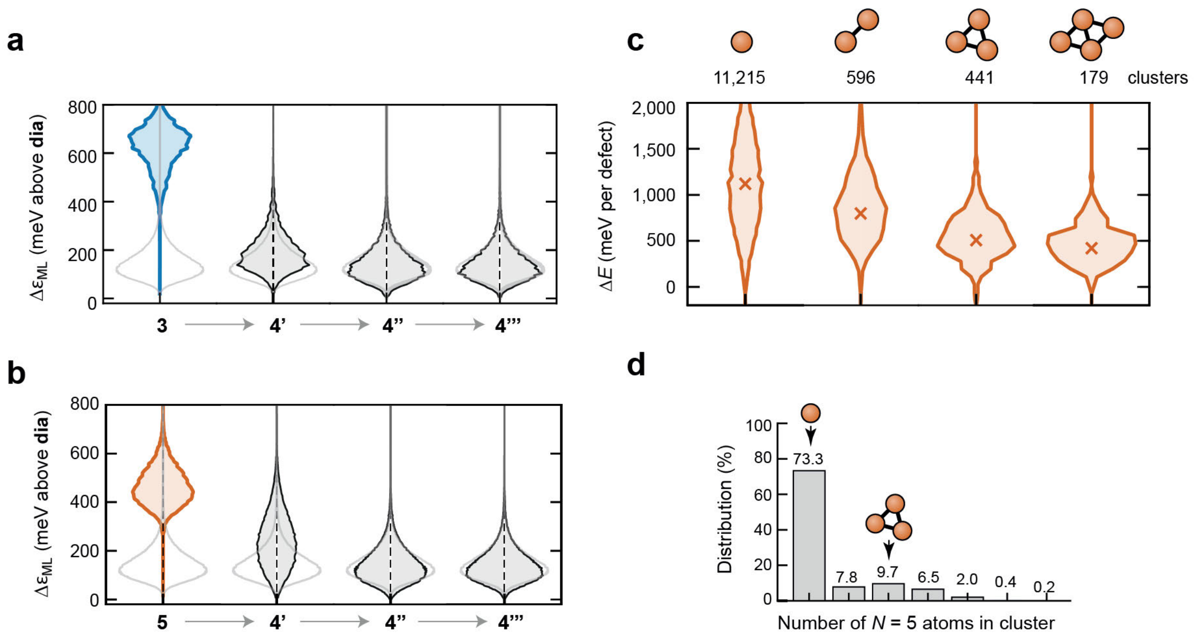

Figure S7. Locality effects in the alternative 1-million atom structure. Further analysis of the locality of defects for the annealed 1-million atom structure of Figure S6. (**a**) Distribution of ML local energies for $N = 3$ atoms and their surroundings. The distribution for bulk a-Si is shown in light grey. (**b**) Same but for $N = 5$ atoms and their surroundings. (**c**) ML-predicted energy distributions for the most common 5-fold defect clusters, with the number of occurrences of each structure directly above each bar. Defect cluster energies are calculated by summing the individual atomic energies of defect cores and their immediate topological neighbors up to 3 bonds away relative to the mean a-Si energy and are reported per-coordination defect: viz. $\Delta E = \sum_i (E_i - \bar{E}) / n_5$ where i indicates the topological classification of atom i and runs over 5, 4′, 4″, and 4‴; and n_5 is the number of 5-fold atoms in the cluster. Crosses indicate the mean of each distribution. (**d**) Statistics for the number of occurrences of clustered 5-coordination defects.

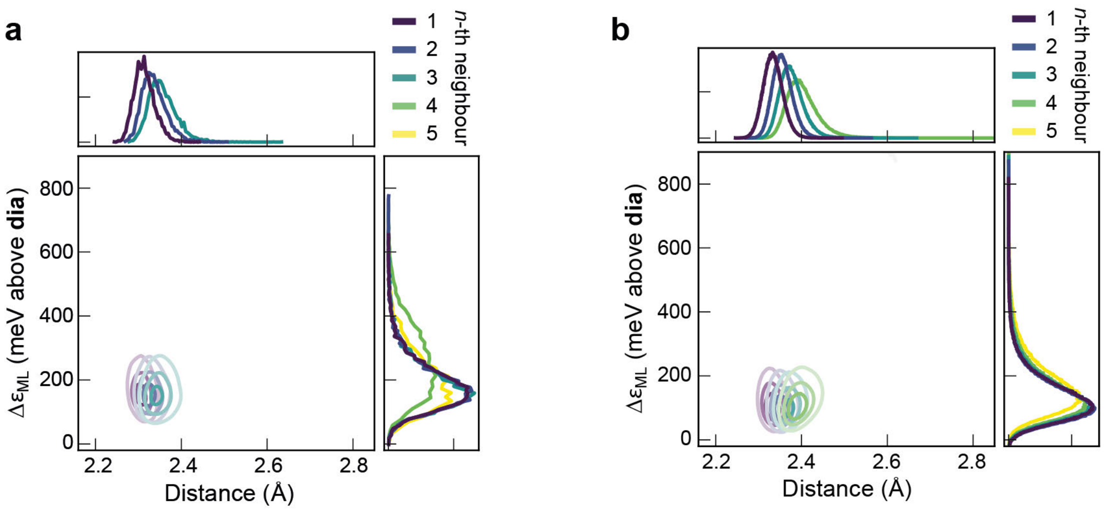

Figure S8. Correlation plots for 3-fold and 4-fold coordinated atoms. Two-dimensional correlation plot of the neighbor density for (**a**) 3-fold defects and (**b**) 4-fold coordinated atoms versus energy, given separately for the immediate neighbors (n = 1–5, purple to green to yellow). Note that the 4-th and 5-th neighbors are off the x-axis scale for 3-fold connected atoms. Likewise for the 5-th neighbors of the 4-fold connected atoms. The energies of 5-th neighbors for 3-fold and 4-fold atoms are like that of bulk a-Si, which indicates that these more distant atoms are typical of the bulk, in contrast to the 5-th neighbors of 5-fold defects.

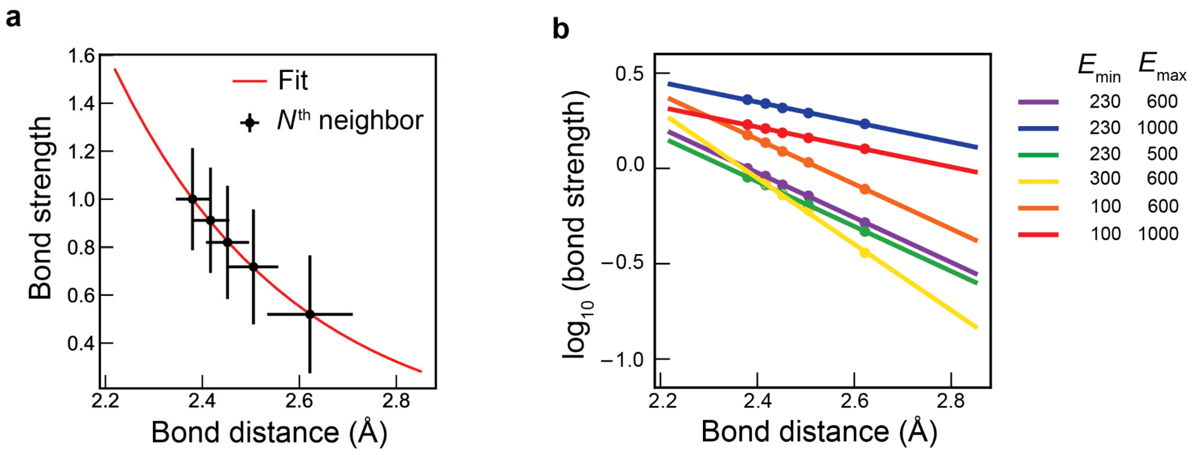

Figure S9. Logarithmic dependence of local-energy-derived bond strength with distance. (**a**) The mean energy (circle) and standard deviation (bars) of each of the Nth neighbors in Figure 1e is fitted to an equation similar to Pauling's relation between atomic radii and interatomic distances in metals[S22]. (**b**) We show fitted equations for a range of energy values for normalization to demonstrate the insensitivity to these values. The modified equation to produce these fits is

$$\log(\, n(E)\,) = A(r_c - r)$$

with the bond strength is defined in terms of local energies as

$$n(E) = \frac{E_{\text{max}} - E}{E_{\text{max}} - E_{\text{min}}}$$

where r_c = 2.38 Å is a characteristic radius (taken to be the mean minimum bond length in the structure), r is the bond distance, and A is a fitting parameter with optimal value 1.17 from linear regression. In the definition of bond strength, we use the approximate highest and lowest energy neighbors of 5-folds to transform local energies to a dimensionless bond-strength measure between 0 (no bond) and 1 (strongest bond). The quality of the fit is not sensitive to the precise values used for E_{min} over the range 100–300 meV and E_{max} over 500–1000 meV. In panel **a**, E_{max} = 600 meV and E_{min}= 230 meV. The sum of the mean bond strengths for the 5 bonds to a fivefold atom is 3.97 using this definition, which suggests that the additional bonds do not make up for their increased weakness.

Directly fitting to the bond-energy data with an additional parameter, B, gives a similar quality of fit using the equation

$$\log(\, E\,) = A(r_c - r) + B.$$

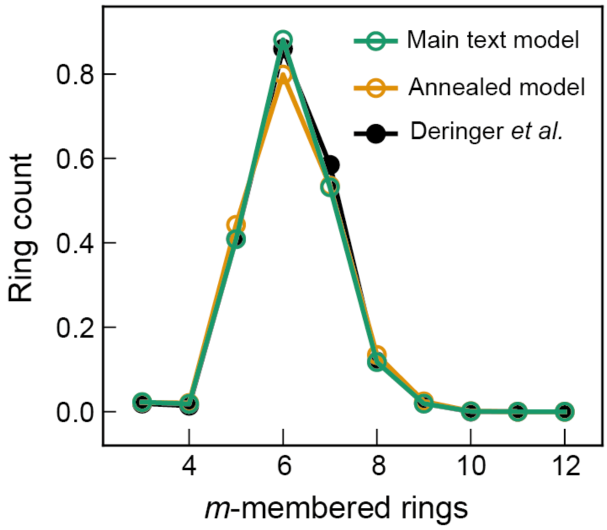

Figure S10. Ring statistics for 1M atom models. Ring counts per atom are displayed for both 1M-atom structural models: the one discussed in the main text (green) and the alternative, newly created annealed structure introduced in Figure S6 (orange). The results are compared to those for a 4,096-atom reference structure (black) from Deringer et al. (Ref. [S8]; obtained with the GAP-18 potential[S4]). The structure discussed in the main text was prepared using the same MD protocol as the 4,096-atom reference and they are here shown to have very similar ring statistics, providing further validation of the quality of the 1M atom model. The algorithm used to obtain ring counts is described in Ref. [S23] (see Computational Methods section above).

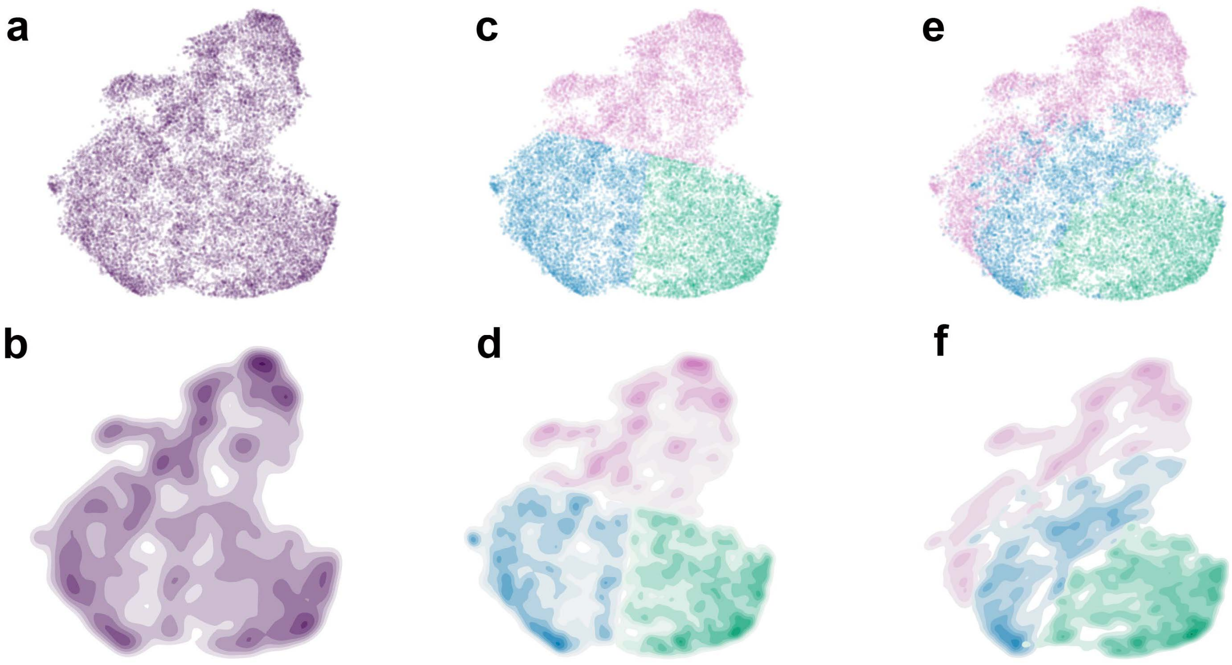

Figure S11. Comparison of clustering methods. Scatter (upper) and gaussian KDE (lower) plots are shown for each of the following: (**a**–**b**) the t-SNE projection of the kernel matrix between SOAP vectors for all 5-fold atoms; (**c**–**d**) coloring by classification label derived from bisecting k-means clustering – an automated method to draw the class boundaries; (**e**–**f**) coloring by classification label derived from comparison with idealized trigonal bipyramidal and floating bond geometries as described in Figure 2. The three most densely populated regions of the map are consistently identified by the supervised and unsupervised classification approaches. The areas throughout the center of the map and in the lower left corner are more uncertain. 74% of environments are put in the same category by both methods.

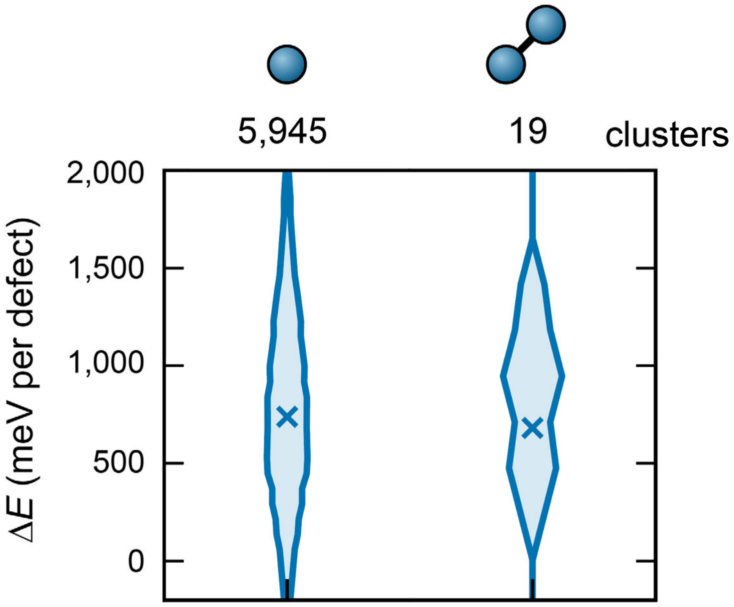

Figure S12. Absence of substantial clustering of 3-fold defects in amorphous silicon. Similar to Figure 5 of the main text, local energy distributions for the largest defect cluster containing only 3-fold atoms are displayed next to the corresponding distribution for an isolated 3-fold defect. As with Figure 5, the defect cluster energies are calculated by summing the individual atomic energies of defect cores and their immediate topological neighbors up to 3 bonds away relative to the mean a-Si energy (excluding other defects) and are reported per-coordination defect. Crosses indicate the mean of each distribution.

Supplementary References

[S1] J. D. Morrow, V. L. Deringer, *J. Chem. Phys.* **2022**, *157*, 104105.

[S2] A. P. Bartók, M. C. Payne, R. Kondor, G. Csányi, *Phys. Rev. Lett.* **2010**, *104*, 136403.

[S3] A. V. Shapeev, *Multiscale Model. Simul.* **2016**, *14*, 1153–1173.

[S4] A. P. Bartók, J. Kermode, N. Bernstein, G. Csányi, *Phys. Rev. X* **2018**, *8*, 041048.

[S5] J. L. A. Gardner, Z. F. Beaulieu, V. L. Deringer, *Digital Discovery* **2023**, *2*, 651–662.

[S6] S. Chong, F. Grasselli, C. Ben Mahmoud, J. D. Morrow, V. L. Deringer, M. Ceriotti, *J. Chem. Theory Comput.* **2023**, *19*, 8020–8031.

[S7] A. P. Thompson, H. M. Aktulga, R. Berger, D. S. Bolintineanu, W. M. Brown, P. S. Crozier, P. J. in 't Veld, A. Kohlmeyer, S. G. Moore, T. D. Nguyen, R. Shan, M. J. Stevens, J. Tranchida, C. Trott, S. J. Plimpton, *Comput. Phys. Commun.* **2022**, *271*, 108171.

[S8] V. L. Deringer, N. Bernstein, A. P. Bartók, M. J. Cliffe, R. N. Kerber, L. E. Marbella, C. P. Grey, S. R. Elliott, G. Csányi, *J. Phys. Chem. Lett.* **2018**, *9*, 2879–2885.

[S9] Y. Pan, F. Inam, M. Zhang, D. A. Drabold, *Phys. Rev. Lett.* **2008**, *100*, 206403.

[S10] D. A. Drabold, Y. Li, B. Cai, M. Zhang, *Phys. Rev. B* **2011**, *83*, 045201.

[S11] P. A. Fedders, D. A. Drabold, S. Klemm, *Phys. Rev. B* **1992**, *45*, 4048–4055.

[S12] N. Bernstein, B. Bhattarai, G. Csányi, D. A. Drabold, S. R. Elliott, V. L. Deringer, *Angew. Chem. Int. Ed.* **2019**, *58*, 7057–7061.

[S13] R. Atta-Fynn, P. Biswas, *J. Chem. Phys.* **2018**, *148*, 204503.

[S14] K. Laaziri, S. Kycia, S. Roorda, M. Chicoine, J. L. Robertson, J. Wang, S. C. Moss, *Phys. Rev. B* **1999**, *60*, 13520–13533.

[S15] R. Xie, G. G. Long, S. J. Weigand, S. C. Moss, T. Carvalho, S. Roorda, M. Hejna, S. Torquato, P. J. Steinhardt, *Proc. Natl. Acad. Sci.* **2013**, *110*, 13250–13254.

[S16] D. A. Drabold, O. F. Sankey, *Phys. Rev. Lett.* **1993**, *70*, 3631–3634.

[S17] V. L. Deringer, N. Bernstein, G. Csányi, C. Ben Mahmoud, M. Ceriotti, M. Wilson, D. A. Drabold, S. R. Elliott, *Nature* **2021**, *589*, 59–64.

[S18] J. Heyd, G. E. Scuseria, M. Ernzerhof, *J. Chem. Phys.* **2003**, *118*, 8207–8215.

[S19] J. Heyd, G. E. Scuseria, M. Ernzerhof, *J. Chem. Phys.* **2006**, *124*, 219906.

[S20] J. Dong, D. A. Drabold, *Phys. Rev. Lett.* **1998**, *80*, 1928–1931.

[S21] L. van der Maaten, G. Hinton, *J Mach Learn Res* **2008**, *9*, 2579–2605.

[S22] L. Pauling, *J. Am. Chem. Soc.* **1947**, *69*, 542–553.

[S23] X. Yuan, A. N. Cormack, *Comput. Mater. Sci.* **2002**, *24*, 343–360.
